# From crop specific to variety specific in crop modeling for the smart farm: A case study with blueberry

**DOI:** 10.1371/journal.pone.0273845

**Published:** 2022-08-30

**Authors:** Gyung Deok Han, Jeong Min Choi, Inchan Choi, Yoonha Kim, Seong Heo, Yong Suk Chung

**Affiliations:** 1 Department of Practical Course Education, Cheongju National University of Education, Cheongju, Republic of Korea; 2 Department of Agricultural Engineering, National Institute of Agricultural Sciences, Jeonju, Republic of Korea; 3 Department of Applied Biosciences, Kyungpook National University, Daegu, Republic of Korea; 4 Department of Horticulture, Kongju National University, Yesan, Republic of Korea; Umeå Plant Science Centre, Umeå University, SWEDEN

## Abstract

Facility cultivation has been evolved from greenhouses to smart farms using artificial intelligence (AI) that simulates big data to maximize production. However, the big data for AI in smart farm is not studied well; the effect of differences among varieties within a crop remains unclear. Therefore, the response of two varieties of blueberry, ‘Suziblue’ and ‘Star’, to light was tested using SAPD meter in order to demonstrate the environmental responses could be different among varieties within the same species. The results showed that those two varieties had significant differences in SPAD values based on the leaf’s position and time, whereas ‘Star’ did not. This indicates that the effect of light depends on the variety, which implies that other traits and other crops may show similar differences. These results are based on a simple experiment. However, it is enough to elucidate that it is extremely important to characterize responses to the environment not only for each crop but also for each variety to collect data for smart farming to increase accuracy for modeling; consequently, to maximize the efficiency of these facilities.

## Introduction

Smart farming is a farm management concept developed to overcome the current challenges of food production caused by the lack of an adequate workforce in rural areas owing to the urban concentration of the population [1]. The focus of smart farming is on the remote control of operations, and the use of artificial intelligence (AI) is envisaged [2,3]. The recent incorporation of AI into the smart farm system requires that crop modeling is performed using big data from appropriate components for the target traits such as yield and quality of crops [4–7]. With the astonishing development in the AI sector accompanied by the design of various remote sensors, the optimized condition can be determined with the given sets of big data. However, there is a limitation in the current system; crop modeling is focused on crops and not varieties within each crop. As each variety comprises mixtures of parents from different genetic and environmental backgrounds, each likely behaves differently in a given environment [8]. This difference could range from small to large and is unknown. However, even a small difference in a single plant may be compounded as large differences when these plants are grown on a large scale for commercial purposes. Unfortunately, this aspect has not yet attracted much attention from researchers, although it is a very important consideration.

The light was selected from among the many environmental factors because it is one of the most variable and significant factors that affects crop growth and yield [9,10]. Blueberry was chosen for the current experiment because it is known as a crop in which the final yield is affected by light conditions [11]. Thus, a simple study on light responses in crop varieties was designed using blueberry, in order to determine whether varieties respond differently to a given environment. Through this study, it is attempted to demonstrate that variety-specific studies are necessary for crop modeling for smart farming.

## Materials and methods

### Plant materials

Blueberry ‘Star’ and ‘Suziblue’ were cultivated in Gosan, Jeju Island. Those two were chosen because they have different genetic background. These cultivars were grown in a rain-shelter house for five years. The SPAD value was measured in 15 replicates at sunrise (8 AM), full sun (1 PM), and sunset (6 PM) for the two cultivars planted in the houses. In addition, SPAD value was measured depending on the position of the foliage directly in contact with sunlight. Leaf position was divided into three classes as high (tree height ratio > 0.9), middle (> 0.5), and low (> 0.1).

### SPAD value

The SPAD-502 chlorophyll meter (Konica Minolta Sensing, Japan) can measure light intensity transmitted through the leaves at two wavelengths (650 and 940 nm) in a non-destructive manner. The SPAD reading value is calculated from the logarithm of the transmittance at 650 nm related to that at 940 nm [12].

### Statistical analysis

Data analysis was performed with the R programming language was used for performing these tests (R v.4.0.4; R Foundation for Statistical Computing, Vienna, Austria). The data sets were checked for normality using the Shapiro-Wilk test [13]. Because some data sets did not show a normal distribution, non-parametric tests (Kruskal-Wallis test) [14] were applied to compare the SPAD value at the three different times (8 AM, 1 PM, and 6 PM) and leaf positions (high, middle, and low).

## Result and discussion

**Fig 1:**
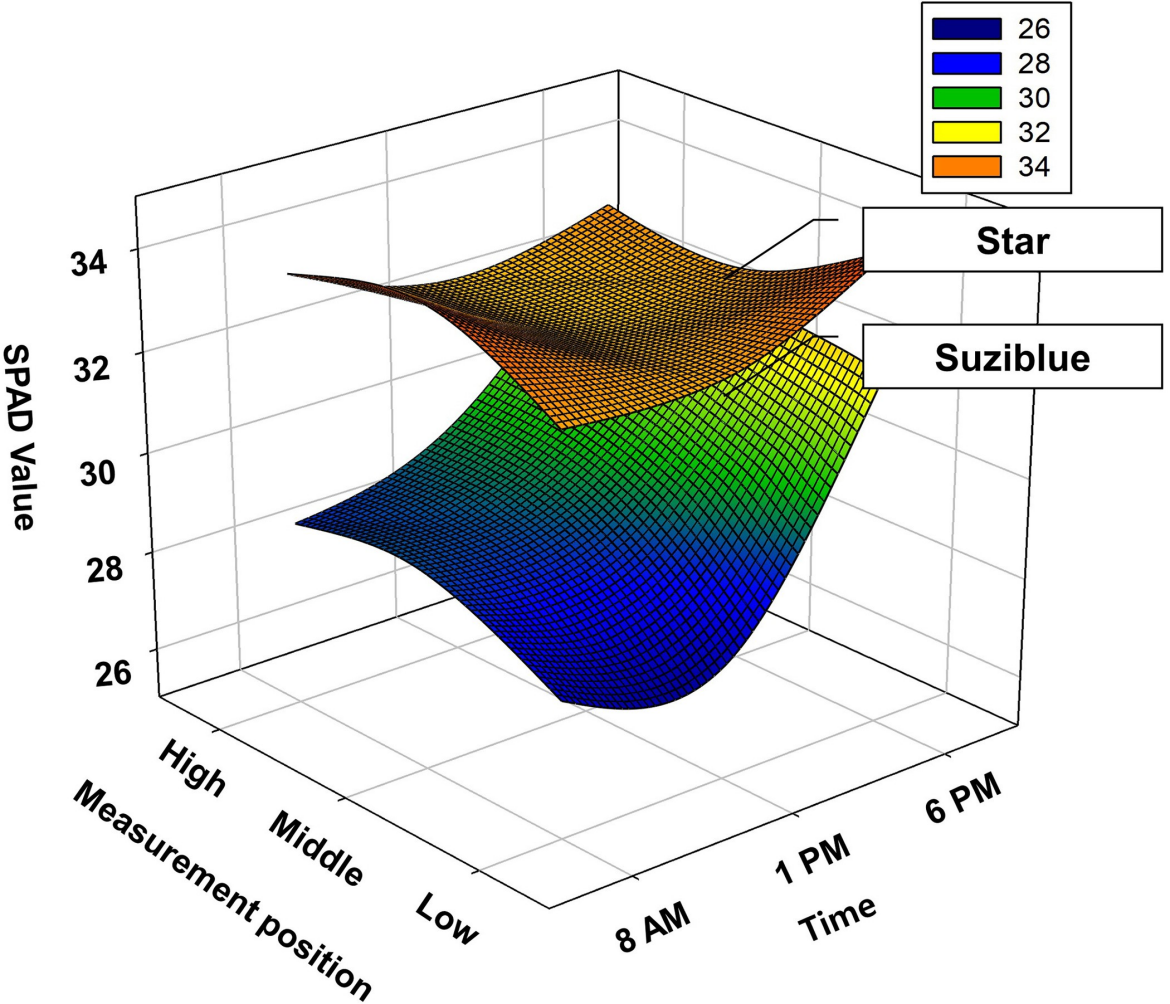
SPAD values of Star and Suziblue at different time and locations.

Significant differences were observed in the SPAD value of the leaves at different times and positions in each cultivar (Table 1). Notably, there was a large difference in SPAD value with respect to time in ‘Suziblue’ compared to that in ‘Star’ (Fig 1). In ‘Suziblue’, both time and leaf position showed effects on the SPAD value; an increasing value was observed in leaves from the low to the high position and with time from early to late.

**Table 1:** P-values from Kruskal-Wallis rank-sum test. ‘Star’ and ‘Suziblue’ were measured at three different times (8 AM, 1 PM, and 6 PM) and three positions (Hight, Middle, and Low).

Parameter	Cultivar		Time		
			8 AM	1 PM	6 PM
SPAD value	Star	Position^a^	0.592 ^NS,^^b^	0.889 ^NS^	0.134 ^NS^
		Rep	0.118 ^NS^	0.224 ^NS^	0.435 ^NS^
	Suziblue	Position	0.183 ^NS^	0.004 **	0.393 ^NS^
		Rep	0.094 ^NS^	0.092 ^NS^	0.076 ^NS^
Parameter	Cultivar		Position		
			High^c^	Middle	Low
SPAD value	Star	Time	0.258 ^NS^	0.118 ^NS^	0.258 ^NS^
		Rep	0.074 ^NS^	0.163 ^NS^	0.330 ^NS^
	Suziblue	Time	9.314e-05 ***	0.001 ***	1.471e-05 ***
		Rep	0.449 ^NS^	0.327 ^NS^	0.783 ^NS^

^a^ df of position and time is 2, df of rep is 14.

^b^ NS, nonsignificant at P > 0.05

**Significant at the 0.01, and

*** Significant at the 0.001.

^c^ High, tree hight ratio > 0.9; Middle, tree hight ratio > 0.5; Low, tree hight ratio > 0.1.

Leaves of the shrub-like blueberry can be divided into sun leaves, and shade leaves based on the position and distribution of the leaves. In general, shade leaves tend to be thick with a thin cuticle and short palisade cells, whereas sun leaves are thin and have a thick cuticle and long palisade cells or form several layers of palisade cells [15]. In the sun leaves, sunlight penetrates the thick mesophyll cells and increases photosynthetic potential. In contrast, shade leaves have evolved to capture the maximum amount of sunlight efficiently for photosynthesis [15]. The photosynthetic efficiency based on the difference in leaf position is because of the differences in the mechanical structure, such as leaf thickness and cell density. Thus, it would be worth examining these traits in the two varieties to investigate the likely cause of this difference. However, whatever the likely cause, this result reveals that there could be differences among varieties in response to sunlight. In contrast, ‘Star’ maintained a high SPAD value at all measured times and leaf positions, although it was slightly lowered at 1 PM when the sunlight was strong.

SPAD value is based on two factors which are the transmission of red light at 650 nm and the transmission of infrared light at 940 nm [16]. Since red light is absorbed by chlorophyll, more precisely chloroplast, while infrared light is not, SPAD could be used to estimate chlorophyll concentration. Indeed, there are studies that show SPAD chlorophyll meter reading can be affected by chloroplast movement [12]. Others also reported that the SPAD value could be changed because of the movement of the chloroplasts that migrate along cell walls perpendicular to the incident light to absorb sunlight to the maximum [17]. However, they also accumulate along cell walls parallel to the direction of the sunlight to avoid absorbing excessive light when exposed to high light levels [18]; consequently, the light transmittance increases under a strong light to decrease the SPAD value. Thus, if the chloroplasts move around corresponding to sunlight direction during the day, the SAPD values could be changed accordingly [12]. However, it could mislead the true chlorophyll concentration. If light intensity changes, the SPAD value would be changed even with the same amount of chloroplasts based on the capacity of light absorbance for photosynthesis [16]. Thus, the SPAD value has to be interpreted carefully. In the current study, the different patterns of SPAD value change during a day. Given the discussion above, there could be two reasons for this observation; chloroplast movement and photosynthetic rate from insensitive photochemical process [19] due to sunlight direction and intensity. However, one thing certain is that the pattern of SPAD values were different in two cultivars implying that the photosynthetic efficiency could be different.

The experiment in the current study had 15 replications per cultivar with no significant difference among those replications based on ANOVA (Table 1). Based on this, the issue of repeatability can be excluded. Then, the left possibility responsible for the different pattern of SPAD values in two cultivars should be the difference of genetic background between them. ‘Suziblue’ was selected from the seedlings of the cross between ‘Star’ and ‘TH-474’, originating from evergreen *Vaccinium darrowii* [20]. Unlike ‘Star’, which reacts immediately to sunlight, ‘Suziblue’ seems not to maximize photosynthetic potential instantaneously despite the increase in light intensity [18]. This appears to be because of the characteristics of *V*. *darrowii*, an evergreen tree in the genome of ‘Suziblue’.

The fact that SAPD values in two different cultivars are different has the following implications for the smart farm system. First, the results can contribute to the decision-making for light addition in the smart farming system. Additional light is necessary for Suziblue, whereas it is not in ‘Star’. This information could be very helpful in reducing costs for setting up a smart farm system. Second, this hypothesis can be applied to other traits in other crops.

Smart farm is evolving with development of artificial intelligence (AI). When AI is applied, it needs big data. However, big data could be useless if it does not fit to statistical analysis which is fundamental of AI; mixture of various data results in low R-squares. This would lead not accurate and not precise results for controlling environments. In the current study, light is one of the most important factors for photosynthesis which is directly associated with yield. The other parameter, time, has different value of light intensity which may result in different responses in two different cultivars. If those factors affect the SPAD value, it could be assumed that it would affect photosynthetic efficacy. Thus, it is here presented that the light responses of different cultivars are different using SAPD values although they are the very same species. There could be due to several reasons such as different genetic background of parents for each cultivar. No matter why it is so, if there is a difference between two varieties within the same species, it should be carefully treated no matter why it is so. Based on this, we suggest that variety-specific studies not crop-specific ones are necessary for crop modeling for smart farming. It would be great that this study will encourage various variety-specific studies for crop modeling to provide the proper big data sets to assist AI operation in a smart farm system.
